# Bolometric-Effect-Based Wavelength-Selective Photodetectors Using Sorted Single Chirality Carbon Nanotubes

**DOI:** 10.1038/srep17883

**Published:** 2015-12-08

**Authors:** Suoming Zhang, Le Cai, Tongyu Wang, Rongmei Shi, Jinshui Miao, Li Wei, Yuan Chen, Nelson Sepúlveda, Chuan Wang

**Affiliations:** 1 Department of Electrical & Computer Engineering, Michigan State University, East Lansing, MI 48824, USA; 2 School of Chemical and Biomolecular Engineering, Nanyang Technological University, Singapore 637459.

## Abstract

This paper exploits the chirality-dependent optical properties of single-wall carbon nanotubes for applications in wavelength-selective photodetectors. We demonstrate that thin-film transistors made with networks of carbon nanotubes work effectively as light sensors under laser illumination. Such photoresponse was attributed to photothermal effect instead of photogenerated carriers and the conclusion is further supported by temperature measurements. Additionally, by using different types of carbon nanotubes, including a single chirality (9,8) nanotube, the devices exhibit wavelength-selective response, which coincides well with the absorption spectra of the corresponding carbon nanotubes. This is one of the first reports of controllable and wavelength-selective bolometric photoresponse in macroscale assemblies of chirality-sorted carbon nanotubes. The results presented here provide a viable route for achieving bolometric-effect-based photodetectors with programmable response spanning from visible to near-infrared by using carbon nanotubes with pre-selected chiralities.

Photodetectors play an important role in modern technology. Generally speaking, the operation of photodetectors can rely on photovoltaic, photo-thermoelectric, or bolometric effects^1^. In particular, bolometers, which are widely used in astronomy and particle physics, transduce the absorbed radiation into a rise in temperature, which in turn induces variations in resistance that can be read out as electrical signals^2^. Over the past two decades, the development in nanomaterials provides new platforms for bolometers with improved sensitivity, speed and flexibility. Semiconducting single-wall carbon nanotubes (sSWCNTs), featured with direct bandgaps and unique one-dimensional van Hove singularities and excitonic states, have been extensively studied for photodetecting applications^3^. An intriguing attribute of sSWCNTs is the structure-dependent bandgap, which enables the wavelength-selective detection of a wide spectrum of light ranging from visible to infrared region. Although a photothermal signature was recently unveiled^4^, the photocurrent of individual sSWCNTs is predominantly photovoltaic in nature, where the incident photons generate strongly bound excitons, which are subsequently separated into free electrons and holes under an electric field^3^. In macroscale assemblies of carbon nanotubes, however, contribution from direct photocurrent can be neglected due to the ultrafast relaxation of excitons and the lack of a sufficient electric field^5^,^6^. Instead, the absorbed energy is effectively transferred to the crystal lattice through strong electron-phonon interaction, which results into a rise in temperature, *i.e*. the bolometric effect^7^. Bolometric response of various carbon nanotube samples, including thin films and polymeric composites, has been demonstrated to be a viable method for photodetection^7^,^8^,^9^,^10^,^11^,^12^,^13^.

Despite the recent progress, all of the reported results are acquired using unsorted carbon nanotubes consisting of both metallic and semiconducting nanotubes with a mixture of all possible chiralities. It is thus very interesting to investigate the bolometric effect of metallic- or semiconducting-enriched and possibly even single chirality carbon nanotubes. A large temperature coefficient of resistance (TCR)—thus a high sensitivity—could be achieved by tuning the ratio of semiconducting and metallic nanotubes in the film^14^,^15^. Due to the extremely sensitive dependence of interband transition energy on chirality in carbon nanotubes, the use of chirality-purified nanotubes could lead to wavelength-selective bolometric response. In fact, the unique optical absorption of SWCNTs have enabled high performance photothermal chromic actuators that are responsive to a programmable range of wavelength^16^, utilizing the recent progress made towards the electronic-type-based or chirality-based sorting and separation of carbon nanotubes in a scalable manner^17^,^18^,^19^,^20^.

Here in this paper, we report the bolometric-effect-based photoresponse in thin-film transistors fabricated using solution-processed sSWCNT networks. The photoresponse (*i.e*. change in resistance) was found to be dependent on the gate voltage applied. For a negative gate voltage, when the device is fully turned on, the carrier mobility decreases due to increased phonon scatterings as the temperature rises upon laser illumination, leading to an increase in resistance. For a positive gate voltage, when the sSWCNT network is depleted, the contribution from increase in carrier concentration at elevated temperatures dominates, resulting in an decrease in resistance. Furthermore, we have demonstrated that by using two types of SWCNT samples with different optical absorption characteristics (a 99% semiconductor-enriched sample and a single chirality (9,8) carbon nanotube sample), the photoresponse exhibits a unique wavelength selectivity, as manifested by the good correlation between the responsive wavelengths of the devices with the absorption peaks of the corresponding carbon nanotubes. This is the first report of controllable and wavelength-selective bolometric photoresponse in macroscale assemblies of chirality-sorted carbon nanotubes.

## Results and Discussion

**Figure 1:**
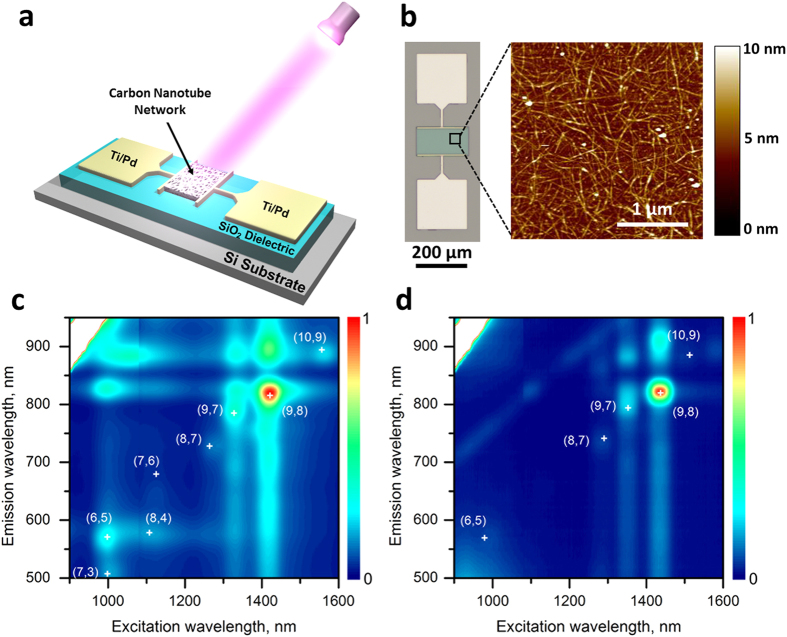
Back-gated thin-film transistor with single chirality (9,8) carbon nanotubes used as a bolometric-effect-based photodetector. (**a**) Schematic diagram of the device. (**b**) Optical microscope image of the transistor (*L* = 100 μm, *W* = 200 μm). Inset: AFM image showing the network of single chirality (9,8) nanotubes in the channel. (**c,d**) Photoluminescence excitation mapping for the (9,8) nanotube sample obtained before (**c**) and after (**d**) the chirality-based enrichment process.

The schematic diagram and optical micrograph of the device (back-gated thin-film transistor) used in this study are presented in Fig. 1a and b, respectively. The fabrication process resembles the methods reported in our previous work^21^,^22^,^23^. Briefly, uniform SWCNT network was deposited onto poly-L-lysine functionalized SiO_2_ surface as the channel semiconductor. After patterning the Ti/Pd source/drain electrodes, the SWCNT thin film, which covers the entire substrate, was patterned using oxygen plasma to define the active channel region. More details of the fabrication process can be found in the Methods section. As shown by the atomic force microscopy (AFM) image in Fig. 1b, the SWCNTs form a dense and uniform network in the channel region, which ensures excellent electrical performance of our devices.

The research and applications of SWCNTs were previously hampered by the difficulties in obtaining high-purity carbon nanotubes with the same electronic type or atomic structure because as-grown SWCNTs usually comprise many possible chiralities that exhibit either metallic or semiconducting behaviors. Fortunately, thanks to the recent progress in post-growth separation, highly purified semiconducting^17^ or even single chirality^18^,^19^,^20^ SWCNTs are now accessible to researchers. In this work, the devices were fabricated using single chirality (9,8) nanotubes prepared through a combination of polymer-assisted extraction^24^ and chirality selective synthesis^25^. The photoluminescence (PL) spectroscopy maps of the (9,8) nanotube sample before and after the chirality-based sorting process are shown in Fig. 1c,d, respectively. From the data, one can easily see that the various semiconducting species in the starting material were effectively removed, leaving behind only (9,8) chirality as the main species, whose purity is estimated to be 73.8% among all semiconducting species (see Table S1 in the Supplementary Information for relative abundance of all semiconducting species calculated based on their PL maps). For comparison purposes, devices with exactly the same structure were also fabricated using 99% semiconducting nanotubes (NanoIntegris, Inc.) similar to those used in our previous work^22^.

**Figure 2:**
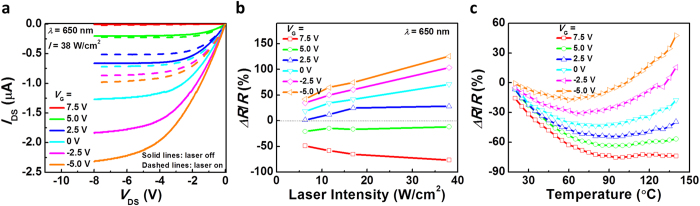
Photoresponse of the single chirality (9,8) nanotube transistor. (**a**) DC output characteristics (*I*_DS_-*V*_DS_) of a (9,8) nanotube transistor with a channel length of 100 μm and a channel width of 200 μm, measured before and after laser illumination (*λ* = 650 nm). (**b**) Relative change in channel resistance as a function of laser intensity for various *V*_G_. (**c**) Relative change in channel resistance as a function of sample temperature for various *V*_G_.

Figure 2a shows the representative output characteristics of a single chirality (9,8) nanotube back-gated thin-film transistor (*L* = 100 μm, *W* = 200 μm) with and without laser illumination. A red laser diode with a wavelength (*λ*) of 650 nm and an intensity of ~38 W/cm^2^ was used. The devices exhibit conventional field-effect-transistor-like output characteristics with clear current saturation. With the laser turned on (dashed lines in Fig. 2a), the output current (*I*_DS_) underwent drastic changes. It can be clearly seen that the changes of *I*_DS_ in response to laser illumination depend on the applied gate voltage (*V*_G_). At *V*_G_ of 5 V and 7.5 V, when the channel is depleted, *I*_DS_ increases slightly upon laser illumination, whereas under more negative *V*_G_, when the transistor is turned on, a significant decrease in *I*_DS_ can be observed.

As with the free standing films of SWCNTs^7^, we attribute the photoresponse in our devices to bolometric effect, *i.e*. a rise in the temperature of SWCNT network upon light irradiation, rather than photovoltaic effect. The conjecture of bolometric effect is reasonable considering the following facts: i) carbon nanotubes possess an extremely high absorption coefficient (10^4^ to 10^5^ cm^−1^) over a wide spectrum extending from ultraviolet to far-infrared region^7^,^26^; ii) carbon nanotubes exhibit strong thermal response to electromagnetic radiation as evidenced by the temperature rise up to 100 K merely due to the environmental blackbody radiation^7^ and ignition phenomenon upon exposure to conventional photographic flashlight^27^; and iii) the monolayer network of SWCNTs in our TFTs has extremely small heat capacity which can lead to a significant increase in temperature due to the absorption of a small amount of energy. In contrast, it is unlikely for the photovoltaic effect (the creation of electron-hole pairs upon light illumination) to be the predominant factor governing the photoresponse in our devices due to a number of reasons: i) With negative gate bias, the current in our device decreases drastically under light illumination, which is contradictory to what is expected from photovoltaic effect; ii) The channel of our device consists of random networks of short carbon nanotubes with numerous tube-to-tube junctions. It will be very difficult, if not impossible, for the photo-generated carriers to reach the electrodes.

In general, the increase in temperature of a semiconductor can result in an increase of carrier concentration due to more thermally excited free carriers as well as a drop in carrier mobility due to increased phonon scattering. Two factors compete with each other in affecting the conductivity of the material. At a very positive *V*_G_, the carrier concentration is rather low because the SWCNT network if fully depleted by the electrostatic coupling from the gate electrode. As a result, the increase in carrier concentration at elevated temperatures upon light illumination would have a stronger influence on the conductivity than the reduction in mobility, resulting in an increased output current. In contrast, as the device transitions into the inversion mode (very negative *V*_G_), the carrier concentration is very high so that the conductivity is dominated by the effect from decrease in carrier mobility.

Figure 2b presents the relative change in channel resistance (*∆R/R*_*0*_), extracted from the output characteristics and plotted as functions of laser intensity for various values of *V*_G_. An unambiguous increasing trend in the magnitude of *∆R/R*_*0*_ can be observed as the laser intensity increases, regardless of the applied *V*_G_. The increase in photoresponse is due to larger temperature changes caused by larger amount of absorbed radiation at higher laser intensities. In addition, the evolution of photoresponse can be clearly identified as *V*_G_ was swept from 7.5 V to −5 V. A transition point (*∆R/R*_*0*_ = 0%) exists somewhere between *V*_G_ = 2.5 V and 5 V (the blue and green lines in Fig. 2b), where the effects from increase in carrier concentration and the reduction in mobility contribute equally to the conductivity. As a result, the output current would ideally remain unchanged irrespective of the laser intensity.

To further support that the observed photoresponse is indeed temperature induced, a single chirality (9,8) nanotube thin-film transistor (*L* = 100 μm, *W* = 100 μm) was heated up to 140 °C using a Peltier heater to study the temperature dependence of its output characteristics (*I*_DS_-*V*_DS_). The channel resistance of the device under various gate biases were then extracted and plotted as a function of temperature as shown in Fig. 2c. The resistance-temperature (R-T) curves show trends that are very similar to the light-induced resistance change shown in Fig. 2b, *i.e*. the channel resistance decreases for positive gate biases (when channel is depleted) and increases for negative gate biases (when channel is turned on). The above provides an unambiguous evidence of the proposed bolometric effects. Furthermore, a close comparison between Fig. 2b,c indicates that the temperature of the carbon nanotube thin-film likely increases drastically when illuminated by laser, which is reasonable considering the high optical absorption and ultralow heat capacity of carbon nanotubes^7^.

Additionally, one can find that at relatively low temperatures, the channel resistance decreases with increasing temperature (negative temperature coefficient of resistance or TCR), showing a semiconductor-like behavior. As the temperature rises further, the TCR gradually turns into positive values, indicating a transition from semiconducting conduction to metallic conduction. The observed R-T relationship can be understood in the framework of a heterogeneous conduction model, in which the carbon nanotube network can be considered as numerous conductive regions separated by barriers. Here, the conductive regions refer to the individual carbon nanotubes or nanotube bundles while the barriers are the inter-tube junctions and intra-tube defects. Consequently, the channel resistance comprises two parts—fluctuation assisted tunneling (FAT) and metallic conduction, which reflect the conduction through the barriers and that inside the conductive regions, respectively. The fact that the TCR changes from negative to positive values implies the transition of primary transport mechanism from FAT to phonon scattering limited conduction. Comparing the R-T curves under different gate biases, one can find that the crossover temperature (when the TCR changes sign) exhibits a significant downshift as the gate voltages decrease from 7.5 to −5 V. In particular, for a gate voltage of 7.5 V, the crossover temperature is likely beyond the temperature range (>140 °C) studied here. Generally speaking, the crossover temperature depends on the carrier concentration, barrier height and width, nanotube length and quality, as well as the overall configuration of the network. In our device, the metallic conduction term plays a more significant role for negative gate biases, when the carrier concentration is high due to electrostatic gating.

**Figure 3:**
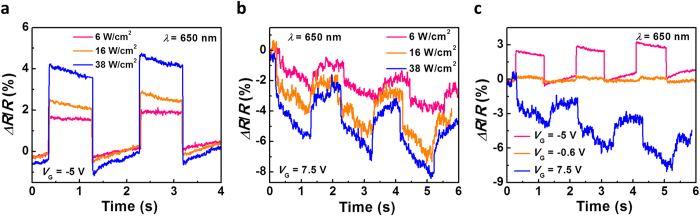
Photo-switching characteristics of the single chirality (9,8) nanotube transistor. (**a**) Time response of the photodetector under different laser intensities of 6, 16 and ~38 W/cm^2^ measured at a *V*_G_ of −5 V. (**b**) Time response of the photodetector under different laser intensity of 6, 16 and 38 W/cm^2^ measured at a *V*_G_ of 7.5 V. (**c**) Time response of the photodetector under a constant laser intensity of 16 W/cm^2^ measured at various *V*_G_ values of −5 V, −0.6 V and 7.5 V.

The photo-switching behaviors measured using the same laser wavelength with different intensities at *V*_G_ = −5 V and *V*_G_ = 7.5 V are shown in Fig. 3a,b, respectively. Higher laser intensities result in larger photoresponse, which is in agreement with the results in Fig. 2b. The response time is estimated to be less than 5 ms at *V*_G_ of  −5 V and 9~11 ms at 7.5 V, both of which are typical values for bolometric effects. Figure 3c compares the photo-switching characteristics measured at different gate voltages with a laser intensity of 16 mW/cm^2^. Again, opposite photoresponse is observed when the device is in “on” (*V*_G_ = −5 V) and “off” (*V*_G_ = 7.5 V) states. Here, we notice that the transition point is at around *V*_G_ of −0.6 V (orange curve in Fig. 3c), where the device exhibit negligible photoresponse. The discrepancy between the transition point and relative resistance change measured from the time response (Fig. 3c) and those deduced from Fig. 2b could be attributed to the influence of gate stress and substrate as the data presented in Fig. 2 was collected significantly longer after the laser was turned on.

**Figure 4:**
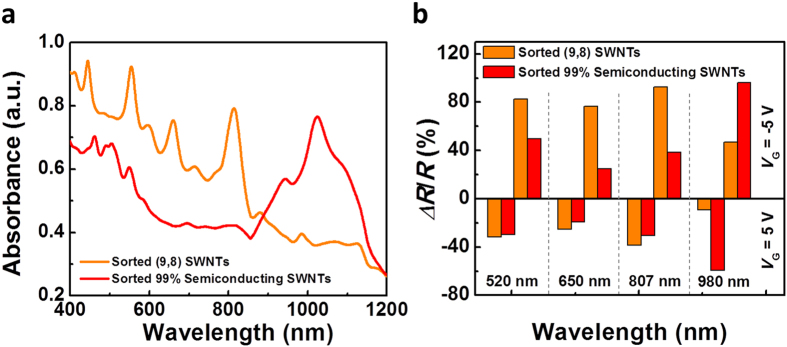
Wavelength-selectivity of the bolometric-effect-based photodetectors with different types of carbon nanotubes. (**a**) UV-Vis-NIR absorption spectra of two different types of SWCNTs, including single chirality (9,8) SWCNTs (orange) and 99% semiconductor-enriched SWCNTs from NanoIntegris, Inc. (red). (**b**) Photoresponse of devices made with the above two kinds of SWCNTs measured under different laser wavelengths of 520, 650, 807, and 980 nm. The histogram in the upper half of the graph corresponds to a *V*_G_ = −5 V and bottom half corresponds to a *V*_G_ = −5 V. Same as panel 4a, the orange columns and red columns correspond to (9,8) SWCNT sample and 99% semiconducting SWCNT sample, respectively.

Finally, we compare the photoresponse of devices fabricated with different types of carbon nanotubes, including the above-discussed (9,8) nanotubes and a 99% semiconducting nanotube sample purchased from Nanointegris, Inc. Figure 4a compares the absorption spectra of these two carbon nanotube samples. The single chirality (9,8) SWCNT sample exhibits several sharp absorption peaks at wavelengths around 555, 660 and 813 nm, whereas the 99% semiconducting nanotube sample from Nanointegris exhibits two broad peaks around 500 and 1024 nm. Both types of devices went through the same measurements performed in Fig. 2 using four laser diodes with different wavelengths, namely, 520 nm, 650 nm, 807 nm, and 980 nm. The photoresponse (*∆R/R*_*0*_) of both types of devices were extracted from the output characteristics (Supplementary Information Figure S2) and summarized in Fig. 4b. It can be seen that the dependence of photoresponse on gate voltage applies to the semiconducting-enriched SWCNTs as well (*i.e*. positive *∆R/R*_*0*_ for negative gate voltages and negative *∆R/R*_*0*_for positive gate voltages). More importantly, the trend in photoresponse for different wavelengths correlates well with the corresponding absorption spectra in Fig. 4a. Specifically, the device with single chirality (9,8) nanotubes (orange color in Fig. 4b) exhibit larger photoresponse than the device with 99% semiconducting nanotubes (red color) at wavelengths of 520, 650 and 807 nm, each of which situates closely to the absorption peaks of (9,8) nanotubes. In contrast, for the laser with a wavelength of 980 nm, the absorption of (9,8) nanotubes become much weaker compared with the 99% semiconductor-enriched nanotube sample, who has a strong and broad absorption peak at around 1024 nm. Not surprisingly, the device with 99% semiconductor-enriched nanotubes exhibit much larger photoresponse at a wavelength of 980 nm compared with the device with single chirality (9,8) nanotubes.

## Conclusion

The results presented here indicate the feasibility of using chirality-sorted SWCNTs for realizing wavelength-selective photodetection based on bolometric effects. Thanks to the abundance of atomic and electronic structures of SWCNTs, there are plenty of options for the design of high performance photodetectors that are sensitive to a preselected range of spectrum throughout the visible to near-infrared region. In addition, the opposite photoresponse under different gate voltages enables another knob of control for additional tunability and adaptability in such programmable photodetectors. It is also worth pointing out that SWCNT network, instead of individual SWCNTs, are used here, which offers the advantage of low cost and scalable fabrication of devices with minimum performance variations using a room temperature solution-based process. On the other hand, due to the bolometric nature of the photoresponse, the influence of environmental factors (substrate, atmosphere, and humidity, *etc*.) can have an effect on the device performance, and it deserves further investigation.

## Methods

### Chirality-based sorting of (9,8) SWCNTs

SWCNTs were synthesized using CoSiO_4_/SiO_2_ catalyst by chemical deposition of carbon monoxide at 6 bar and 780 °C. This catalyst shows good chiral selectivity toward (9,8) nanotubes. The details of catalyst preparation and synthesis conditions were described in our previous publication^25^. As-synthesized samples were refluxed in 1 M NaOH aqueous solution for 2 h to remove SiO_2_ substrate. The extraction of (9,8) SWCNTs was carried out using poly[(9,9-dihexylfluorenyl-2,7-diyl)-co-(9,10-anthracene)] (PFH-A) (American Dye Source Inc.)^24^. SWCNT powders (2 mg) and polymers (15 mg) were added into toluene (20 mL). The suspension was first homogenized in a sonic bath (Fisher, elmasonic S60H) for 1 h at 0 8C, and then tip-sonicated for 10 min. Afterwards, the suspension was centrifuged at 50000 g for 1 h to obtain a clear supernatant. The supernatant was then used direclty for characterization and device fabrication. As a reference for SWCNT charactersization, SWCNTs were also dispersed in 2% (w/v) aqueous SDBS solution. SWCNTs (1 mg) were added into sodium dodecylbenzene sulfonate (SDBS) solution (10 mL), and the suspension was sonicated by a tip sonicator (SONICS, VCX-130) for 1 h at 20 W in an ice bath. Then, the suspension was centrifuged at 50000 g (Hitachi-Koki, CS150GXL) for 1 h. PL spectroscopy maps of SWCNT solutions were measured using a spectrofluorometer (Jobin-Yvon Nanolog-3) with the excitation scanned from 500 to 950 nm and the emission collected from 900 to 1600 nm.

### Device fabrication

Firstly, Poly-L-Lysine solution (0.1% w/v in water, Sigma Aldrich) was used to functionalize the Si/SiO_2_ surface for 5 min, followed by immersing the samples into 0.01 mg/ml sorted single chirality (9,8) SWCNT solution or commercially available 99% semiconducting SWNTs solution (NanoIntegris Inc.) for 5 min. The resulting SWCNT films in both samples had similar network density (between 30 to 40 tubes/μm^2^). Source and drain electrodes (0.5 nm Ti and 50 nm Pd) were then patterned using photolithography, electron beam evaporation, and lift-off process. The heavily-doped silicon substrate serves as the back gate. As a final step, the nanotubes outside the active channel region were patterned and etched away using oxygen plasma.

### Electrical and photoresoponse measurements

All electrical measurements were carried out using a Signatone probe station and an Agilent B1500A semiconductor parameter analyzer. Laser diodes (Osram PLP520-B1 for 520 nm, LPC-826 for 650 nm, Thorlabs L808P200 for 808 nm, and Thorlabs L980P010 for 980 nm) were mounted onto the probe station and focused onto the sample. The laser power was controller by a DC power supply (Keithley 2450 Sourcemeter) in current source mode and measured using an optical power meter (Thorlabs PM100D and S121C). The size of the laser beam spot was intentionally made large enough to cover the whole channel region (100 × 200 μm^2^) of the device. The laser beam was projected onto a white paper with printed 1 mm × 1 mm grids and captured with a CCD camera mounted on the microscope for estimating the beam size and calculating the laser intensity. For the temperature measurement, the samples were attached to a Peltier heater, whose temperature was precisely controlled by a temperature controller (Thorlabs TED4015).

## Additional Information

**How to cite this article**: Zhang, S. *et al*. Bolometric-Effect-Based Wavelength-Selective Photodetectors Using Sorted Single Chirality Carbon Nanotubes. *Sci. Rep*. **5**, 17883; doi: 10.1038/srep17883 (2015).

## Supplementary Material

Supplementary Information
