# Supplementary material for: Bolometric-Effect-Based Wavelength-Selective Photodetectors Using Sorted Single Chirality Carbon Nanotubes
Source: Sci Rep. 2015 Dec 8;5:17883. doi: 10.1038/srep17883 (PMC4672266; doi:10.1038/srep17883)
Supplement: Supplementary Information [file srep17883-s1.doc]

**Bolometric-Effect-Based Wavelength-Selective Photodetectors Using Sorted Single Chirality Carbon Nanotubes**

*Suoming Zhang^1^,* ***^§^****, Le Cai^1^****^,§^****, Tongyu Wang^1^, Rongmei Shi^2^, Jinshui Miao^1^, Li Wei^2^, Yuan Chen^2^, Nelson Sepúlveda^1^, and Chuan Wang^1*^*

*^1^Department of Electrical & Computer Engineering, Michigan State University, East Lansing, MI 48824*

*^2^School of Chemical and Biomolecular Engineering, Nanyang Technological University, Singapore 637459*

**Corresponding author:* [*cwang@msu.edu*](mailto:cwang@msu.edu)

***^§^****These authors contributed equally to this work*

**Supplementary Information**

**S1. Relative abundance of all semiconducting species.**

The relative abundance was estimated based on the photoluminescence intensity of individual (n,m) species.

| (n,m) | *d_t_*,nm | λ_11_, nm | λ_22_, nm | Abundance, % | |
| --- | --- | --- | --- | --- | --- |
|  |  |  |  | Unsorted | Sorted |
| (6,4) | 0.692 | 873 | 578 | 3.2 |  |
| (6,5) | 0.757 | 976 | 566 | 12.3 | 4.9 |
| (7,3) | 0.706 | 992 | 504 | 6.8 |  |
| (7,6) | 0.895 | 1120 | 648 | 2.9 |  |
| (8,4) | 0.840 | 1111 | 589 | 5.7 |  |
| (8,7) | 1.032 | 1265 | 728 | 4.3 | 5.1 |
| (9,7) | 1.103 | 1322 | 793 | 14.2 | 11.5 |
| (9,8) | 1.170 | 1410 | 809 | 41.4 | **73.8** |
| (10,9) | 1.307 | 1556 | 889 | 9.2 | 4.7 |

**Table S1.** Relative abundance of major semiconducting nanotubes in the unsorted and sorted single-wall carbon nanotube samples.

**S2. Photoresponse of two types of nanotube transistors under laser illumination with different wavelengths.**

As shown in the figure below, the photoresponse of the devices varies with serval factors including the laser wavelength used, gate voltage applied, and the type of carbon nanotubes used. So is the responsivity. For the single chirality (9,8) carbon nanotubes, the highest responsivity is around 3.68×10^-4^ A/W. For the 99% semiconducting SWNTs, the highest responsivity is around 7.36×10^-3^ A/W.


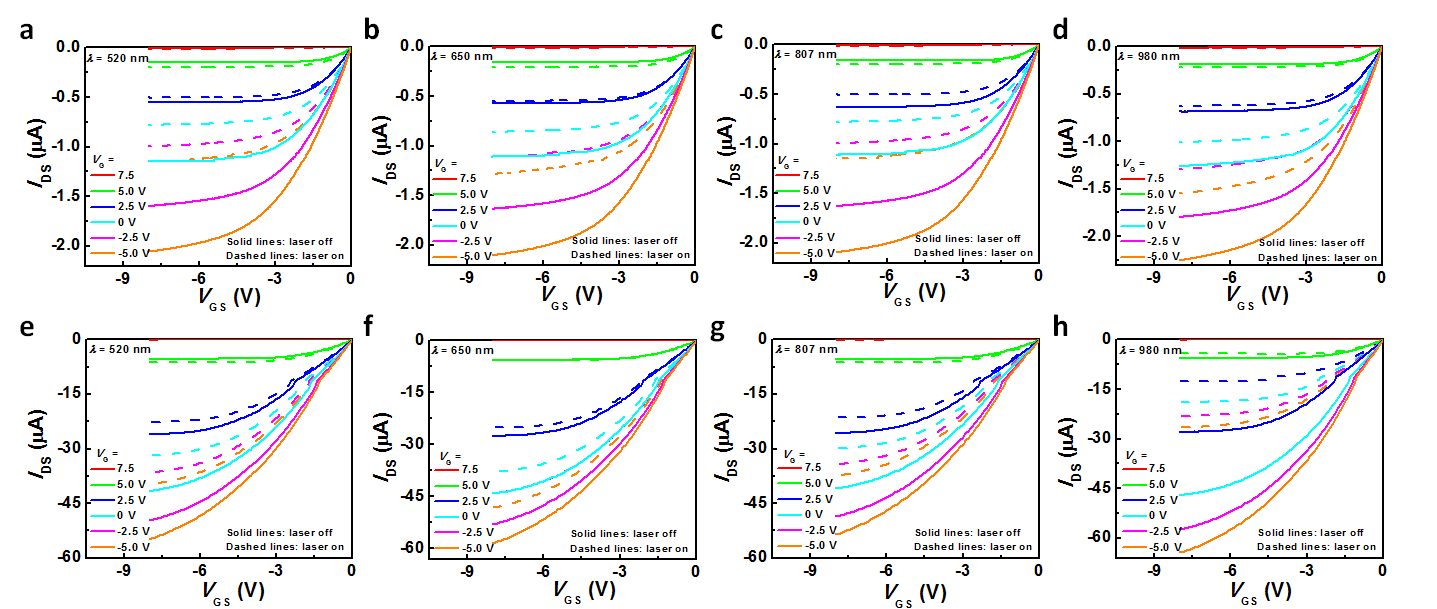


**Figure S2.** (a-d) Output characteristics (*I*_DS_-*V*_DS_) of a (9,8) nanotube thin-film transistor measured before and after laser illumination with (a) *λ* = 520 nm, (b) *λ*=650 nm, (c) *λ*=807 nm, and (d) *λ*=980 nm. (e-h) Output characteristics of a 99% semiconducting nanotube thin-film transistor measured before and after laser illumination with (e) *λ* = 520 nm, (f) *λ*=650 nm, (g) *λ*=807 nm, and (h) *λ*=980 nm.
